# Effects of repeated trials on the strategy used for a hand laterality judgment task

**DOI:** 10.1007/s00221-025-07166-w

**Published:** 2025-09-29

**Authors:** Kohei Onishi, Kotaro Takeda, Kenji Kato, Yutaka Sato, Nobuaki Shimoda

**Affiliations:** 1 Graduate School of Health Sciences, Fujita Health University, Toyoake, Japan; 2 Faculty of Rehabilitation, School of Health Sciences, Fujita Health University, Toyoake, Japan; 3 Laboratory for Clinical Evaluation with Robotics, Assistive Robot Center, National Center for Geriatrics and Gerontology, Obu, Japan; 4 Graduate School of Technology, Industrial and Social Sciences, Tokushima University, Tokushima, Japan; 5 Department of Rehabilitation, Faculty of Health Sciences, Tokyo Kasei University, Sayama, Japan

**Keywords:** Hand mental rotation, Implicit motor imagery, Reaction time, Biomechanical constraints effect, Changes in performance strategy

## Abstract

The hand laterality judgment task requires participants to determine whether a picture of a hand, presented at various rotational angles, depicts a left or right hand. Several strategies have been suggested to be involved in task performance: in particular, palm-view pictures are thought to rely on motor imagery (MI), whereas back-view pictures are thought to rely on ‘nonMI’ (i.e., without motor imagery) strategies, including visual imagery (VI). However, the influence of repeated task execution on performance strategies remains unclear. This study examined the relationship between self-reported strategies and response time (RT) profiles during a 512-trial hand laterality judgment task in 42 healthy adults. Based on post-task self-reports for palm-view pictures, participants were classified into the MI group, consistently using MI throughout the trials, and the MI–nonMI group, switching from MI to nonMI during the repeated trials. In the MI group, RT profiles consistently showed longer RTs for lateral palm-view pictures (outward-pointing fingers) than for medial orientations (inward-pointing fingers), characteristic of MI use, across both halves of the task. The MI–nonMI group showed similar RT patterns initially, but in the second half, RT differences between lateral and medial orientations diminished, suggesting a shift toward VI-like characteristics. These findings suggest that although both groups may have used MI, RT trends varied according to the participants’ self-reported strategies. In the MI group, both explicit self-report and implicit RT profiles indicated sustained MI use, whereas the MI–nonMI group, self-reports indicated a strategy shift to nonMI, and their RT profiles suggest a combined use of MI and nonMI.

## Introduction

A mental rotation task is generally defined as a cognitive task that requires judging whether two figures presented at different angles are identical or mirror images of each other. In such figure comparison tasks, response time (RT) is typically proportional to the angular difference between the two figures, suggesting that participants mentally rotate the presented figures while performing the task (Shepard and Metzler 1971). One type of mental rotation task is the hand laterality judgment (HLJ) task, in which participants are required to judge whether a picture of a hand presented at various orientations corresponds to the left or right hand. In the HLJ task, a major performance strategy is motor imagery (MI), in which participants implicitly simulate their own hand movements to align with the presented picture (Parsons 1987). Evidence supporting MI involvement comes from the medial–lateral (ML) effect: RTs are shorter when the fingertips of a palm-view hand picture point medially (toward the body) and longer when they point laterally (away from the body), reflecting biomechanical constraints (Sekiyama 1982; Parsons 1994; Takeda et al. 2010; Bläsing et al. 2013; Dalecki et al. 2013). The ML effect has thus been regarded as behavioral evidence of MI engagement and is most consistently observed for palm-view pictures (Vannuscorps et al. 2012; De Simone et al. 2013; Zapparoli et al. 2013; Conson et al. 2017; Nagashima et al. 2019, 2021). Recent studies further confirmed that this effect is primarily linked to palm-view pictures using computational modeling (Moreno-Verdú et al. 2025b) and demonstrated its robustness even in online experimental settings, where participants’ postures are less controlled (Moreno-Verdú et al. 2025a). By contrast, back-view pictures typically yield RTs that vary mainly with angular disparity from the upright position, regardless of biomechanical plausibility. This pattern is usually interpreted as reflecting strategies other than MI, such as visuospatial imagery (often termed VI) or heuristic use of salient cues like thumb–index orientation (Parsons 1987; ter Horst et al. 2010; Dahm and Draxler 2023). Importantly, the terminology for these strategies has been inconsistent across studies. For instance, our previous studies (Nagashima et al. 2019, 2021) used “motor imagery” to specifically denote a kinesthetic strategy, contrasted with “visual imagery,” whereas other authors have defined a broader “motor strategy” that incorporates both kinesthetic and visual MI, contrasted with a “visual strategy” based on visual transformation (Conson et al. 2020), as summarized by Bek et al. (2022). To avoid such ambiguity, in the present study we collectively define these non-motoric approaches as nonMI strategies, referring to strategies that do not involve simulating one’s own movement and are less likely to produce an ML effect, in contrast to MI strategies that are characterized by the influence of biomechanical constraints.

Studies have reported that variations in performance strategies within the HLJ task are not solely attributable to the distinction between palm and back-view pictures. For example, RTs increased when a human silhouette was displayed behind a palm picture with the fingertips pointing medially (Conson et al. 2012). This finding suggests that the human silhouette interfered with first-person MI, prompting a shift toward a visual perspective strategy. In another study, RTs increased when the thumb was fixed in an adducted position for 1 h prior to the task (Bläsing et al. 2013). Similarly, RTs were longer when participants placed their hands behind their backs compared with when their hands were placed on their knees, suggesting that hand position during the task may influence the initial posture adopted for MI (Ionta and Blanke 2009; Ionta et al. 2012). In addition, performance strategies vary with age and individual characteristics (Nagashima et al. 2019, 2021), which can in turn influence task performance, as represented by the overall mean RT across all pictures. Specifically, Nagashima et al. (2021) found that palm-view pictures elicited MI across all ages and abilities, whereas for back-view pictures, younger participants predominantly used VI, while middle-aged and elderly participants tended to use VI if they were high performers and MI if they were low performers.

In the present study, we hypothesized that the number of task repetitions in the HLJ task could also influence RTs and induce changes in performance strategies. Repeated execution of actual movements may lead to more efficient response preparation, such as determining direction and force, thereby reducing overall movement time (Mawase et al. 2018). Similarly, repetition tasks involving calculation or working memory result in reduced completion times and improved accuracy, with changes in performance strategies considered a contributing factor (Jonides 2004). Comparable effects of task experience have also been reported for visual rotation tasks, such as mental paper folding (Dahm and Draxler 2023), and for the mental body rotation task (Dahm et al. 2022), whose Supplementary Material reports learning effects in RTs and strategy use. Although many studies have suggested that participants primarily use MI in the HLJ task, particularly for palm-view hand pictures (Ionta and Blanke 2009; ter Horst et al. 2012; Zapparoli et al. 2014; Conson et al. 2017; Nagashima et al. 2021), MI is not necessarily required (Mibu et al. 2020). A notable difference between studies lies in the number of picture repetitions: studies supporting the use of MI typically present each hand picture 2–6 times to calculate average RTs, whereas Mibu et al. presented each picture 12 times.

To address this issue, the present study used post-task self-reports and RT profiles to investigate whether the number of repetitions in the HLJ task influences performance strategies. Specifically, participants were classified on the basis of their self-reported strategies, and changes in RTs and the ML effect (calculated from RTs) across repeated trials were analyzed.

## Methods

### Participants

The participants were 42 right-handed individuals (20 males, 22 females; mean age, 25 ± 1.6 years), none of whom had a history of orthopedic or central nervous system disorders affecting the upper or lower extremities. Handedness was assessed using the Edinburgh Handedness Inventory (Oldfield 1971). The objectives and methods of the study were explained to participants both verbally and in writing. All participants provided written informed consent. The study was conducted in accordance with the Declaration of Helsinki and was approved by the ethics committee of Fujita Health University.

### Environment and experimental procedures

**Fig. 1:**
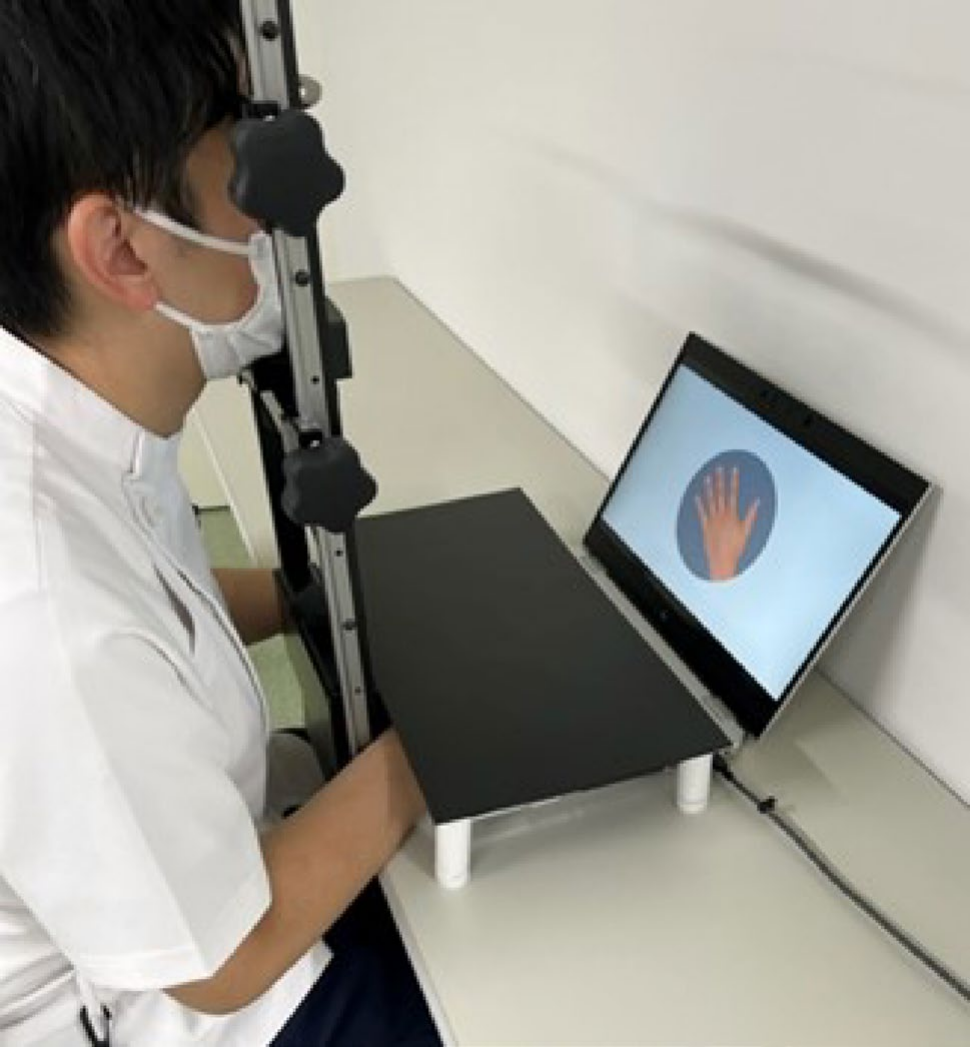
Experimental environment. Participants’ head tilt was restrained using a chin rest during both the left–right choice task and the hand laterality judgment task. Their hands were covered with a masking board to prevent them from using the direction of their own hands as a reference. They discriminated between the left and right sides of the displayed arrow and hand images

The participants were seated in a quiet room on a chair positioned in front of a 15.6-inch laptop computer (EliteBook 1050 G1; Japan HP, Tokyo, Japan), with their heads stabilized using a chin rest approximately 60 cm away from the display (Fig. 1). They placed their left and right index fingers on the “F” and “J” keys, respectively, of the keyboard and performed two experimental tasks: a left–right choice task using arrow pictures and the HLJ task using hand pictures. Before beginning these tasks, participants completed a practice session using four arrow pictures and four hand pictures to familiarize themselves with the procedures. They then performed the left–right choice task, followed by the HLJ task.

**Fig. 2:**
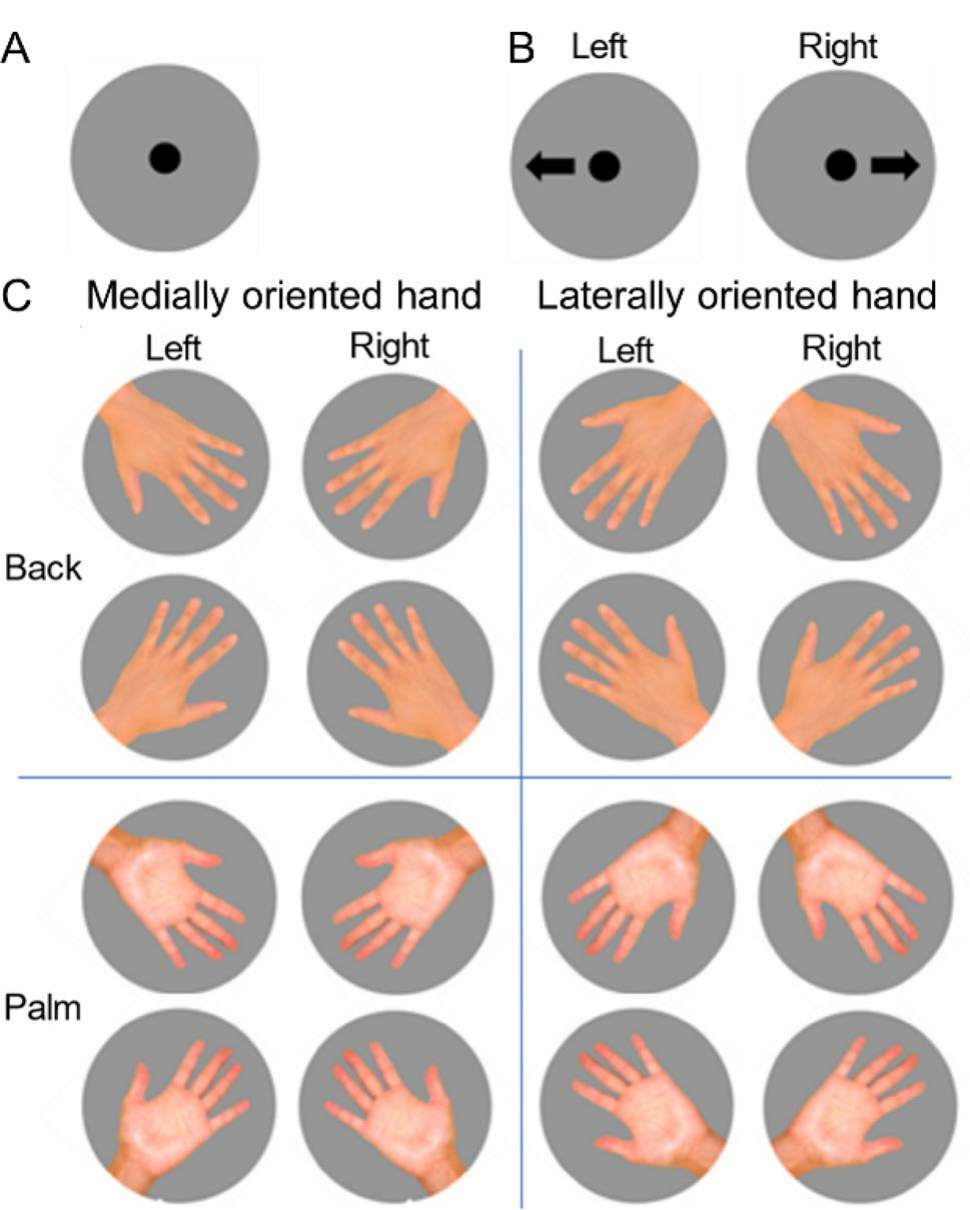
Pictures of hands and arrows used in the present experiment. The left–right choice task and the hand left–right judgment task were conducted separately. Following the presentation of the fixation point (**A**), a single arrow picture for the left–right choice task (**B**) or a single hand picture for the left–right judgment task (**C**) was presented randomly, one at a time. These pictures were identical to those in a previous study (Nagashima et al. 2021). “Medially oriented hand” refers to pictures with the fingertips facing inward toward the body, whereas “laterally oriented hand” refers to pictures with the fingertips pointing outward, away from the body

In both tasks, a fixation point (Fig. 2A) was displayed for 2 s at the beginning of each trial, followed by a picture (a single arrow picture for the left–right choice task or a hand picture for the HLJ task). Participants were instructed to judge as quickly and accurately as possible whether the arrow pointed left or right, or whether the hand picture depicted a left or right hand, by pressing the “F” key for left and the “J” key for right. Once the participant responded, the picture disappeared, and the fixation point was displayed again for 2 s before the next picture appeared. The presentation of pictures, as well as the measurement of RT and accuracy, was controlled using E-prime 3.0 (Psychology Software Tools, Inc., Pittsburgh, PA, USA).

In the left–right choice task, left- and right-pointing arrow pictures (Fig. 2B) were each presented 16 times in random order, for a total of 32 trials. In the HLJ task, each trial set consisted of 16 different hand pictures (palm/back × left/right × 4 orientations; Fig. 2C), each presented once in random order. Participants completed 32 such trial sets consecutively, without breaks between sets, resulting in 512 trials in total. Within each HLJ trial set, the order of pictures was randomized, and the presentation order was adjusted so that the last picture of one set did not appear as the first picture of the following set.

After completing the HLJ task, participants recalled and reported their strategy for distinguishing between left and right hands separately for palm-view and back-view pictures, based on their memory of performing the corresponding trials. Written responses were obtained in an open-ended format. If these responses did not indicate whether the same strategy was used consistently or changed during the task, the examiner conducted a brief verbal interview to clarify this point. Based on the self-reports for the palm-view pictures, the participants were categorized into the following four groups: (1) the MI group, which performed the HLJ task consistently using the MI strategy (imagining their own hand movement); (2) the nonMI group, which performed the task without the MI strategy; (3) the MI–nonMI group, which initially used MI but later switched to nonMI strategy; and (4) the nonMI-MI group, which initially did not use MI but began using it later.

### Data analysis

For both tasks, RTs exceeding 6,000 ms were excluded from the statistical analyses. The cutoff value was chosen to balance the exclusion of excessively long RTs with the need to retain sufficient and evenly distributed trials within each trial block of the HLJ task. This criterion is in line with previous HLJ studies, which have used varying cutoffs (Saimpont et al. 2009; ter Horst et al. 2010; Nagashima et al. 2021). In the left–right choice task, the average RT in the last eight trials for each of the left- and right-pointing arrows was calculated. This value was defined as the motor response generation time, representing the baseline time to execute simple hand movements after performance had stabilized. To standardize the HLJ task RTs and minimize the influence of differences in simple hand movements between the left and right hands, we calculated ΔRT by subtracting the motor response generation time from the RTs of the corresponding hand (Zapparoli et al. 2016; Nagashima et al. 2017, 2019, 2021; Mochizuki et al. 2019). Each picture was presented in 32 trials, which were divided into the first and second 16 trials. The mean ΔRT and accuracy were calculated separately for each half, and the inverse efficiency score (IES) (Townsend and Ashby 1978) was then calculated for each picture for each half (16 trials) by dividing the mean ΔRT by the corresponding accuracy (Nagashima et al. 2021). Each picture was classified on the basis of the hand view (back or palm) and hand orientation (medially oriented hand: fingertips pointing inward, toward the body; laterally oriented hand: fingertips pointing outward) (Fig. 2C). The IESs were then averaged separately for each of the four conditions: back–medially oriented, back–laterally oriented, palm–medially oriented, and palm–laterally oriented hands.

Participants with a correct response rate of < 50% for any of the 16 hand pictures (*n* = 2) and those who fell asleep during the task (*n* = 2) were excluded from the analysis. After the HLJ task, participants were interviewed about their performance strategies for both the palm-view and back-view pictures, and were initially classified into four groups on the basis of their responses. However, only two of these groups, classified by the strategy used for the palm-view pictures, had sufficient sample sizes for statistical analysis: the MI group (*n* = 18) and the MI–nonMI group (*n* = 18). Therefore, only these two groups were included in the following statistical analyses, which examined the ML effect for both palm-view and back-view pictures. Although the experimental design could be represented with four factors (strategy, period, orientation, and view (palm vs. back)), in line with previous studies (Saimpont et al. 2009; Nagashima et al. 2019, 2021) the analyses were conducted separately for palm-view and back-view pictures.

To evaluate the ML effect, separate analyses of variance (ANOVAs) were conducted for palm-view and back-view pictures as follows. For all ANOVAs, when a significant interaction was observed, follow-up analyses were conducted to clarify the nature of the interaction. These analyses involved breaking down higher-order interactions into their simple constituent effects.

First, a three-way ANOVA was performed on the IESs, with strategy (MI group vs. MI–nonMI group) as a between-subjects factor and period (first half vs. second half) and orientation (medially vs. laterally oriented hands) as within-subjects factors. In the context of this three-way model, we tested the interaction between period and orientation within each strategy group. When this interaction was significant, we further analyzed the simple–simple main effects, that is, the effect of orientation within each level of period and the effect of period within each level of orientation. When the simple two-way interaction was not significant, we reported the overall effects of period (first vs. second half) and orientation (medially vs. laterally) without stratifying by the other factor.

In addition, to quantify the magnitude of the ML effect (i.e., the difference in IESs between laterally and medially oriented hands) and to examine how it changed over time, a two-way ANOVA was conducted, with strategy (MI group vs. MI–nonMI group) as a between-subjects factor and period (first half vs. second half) as a within-subjects factor. In the context of this two-way model, we use the term “simple main effects” to denote testing the effect of period within each strategy group, and conversely, the effect of strategy within each period.

## Results

The participants’ self-reported strategies for the palm-view and back-view pictures are listed in Table 1. For the palm-view pictures, 36 out of 38 participants were classified into either the MI or MI–nonMI group, which were included in the following statistical analyses of the IESs. For the back-view pictures, all participants except one were classified into the nonMI group. These descriptions of the strategies are based directly on participants’ written self-reports and, when necessary, supplemented by brief verbal interviews for clarification. As an example, participants in the MI group reported mentally simulating the movement of their own hand to align it with the orientation of the hand shown in the picture. In contrast, participants in the MI–nonMI group initially employed the MI strategy but later switched to the nonMI strategy. Specifically, they used visual cues, such as the orientation of the thumb and index finger or the L-shape formed by them, as an initial cue to infer hand laterality. They then mentally rotated the picture to a more familiar orientation in order to finalize their left–right judgment.

**Table 1:** Explicit strategies for palm- and back-view pictures

Strategy	Palm (n)	Back (n)
MI	18	1
nonMI	1	37
MI–nonMI	18	0
nonMI–MI	1	0

The explicit strategies reported after the HLJ task are listed. MI, motor imagery throughout; nonMI, no use of MI; MI–nonMI, switched from MI to nonMI; nonMI–MI, switched from nonMI to MI. One participant used nonMI for palm-view but MI for back-view pictures

**Fig. 3:**
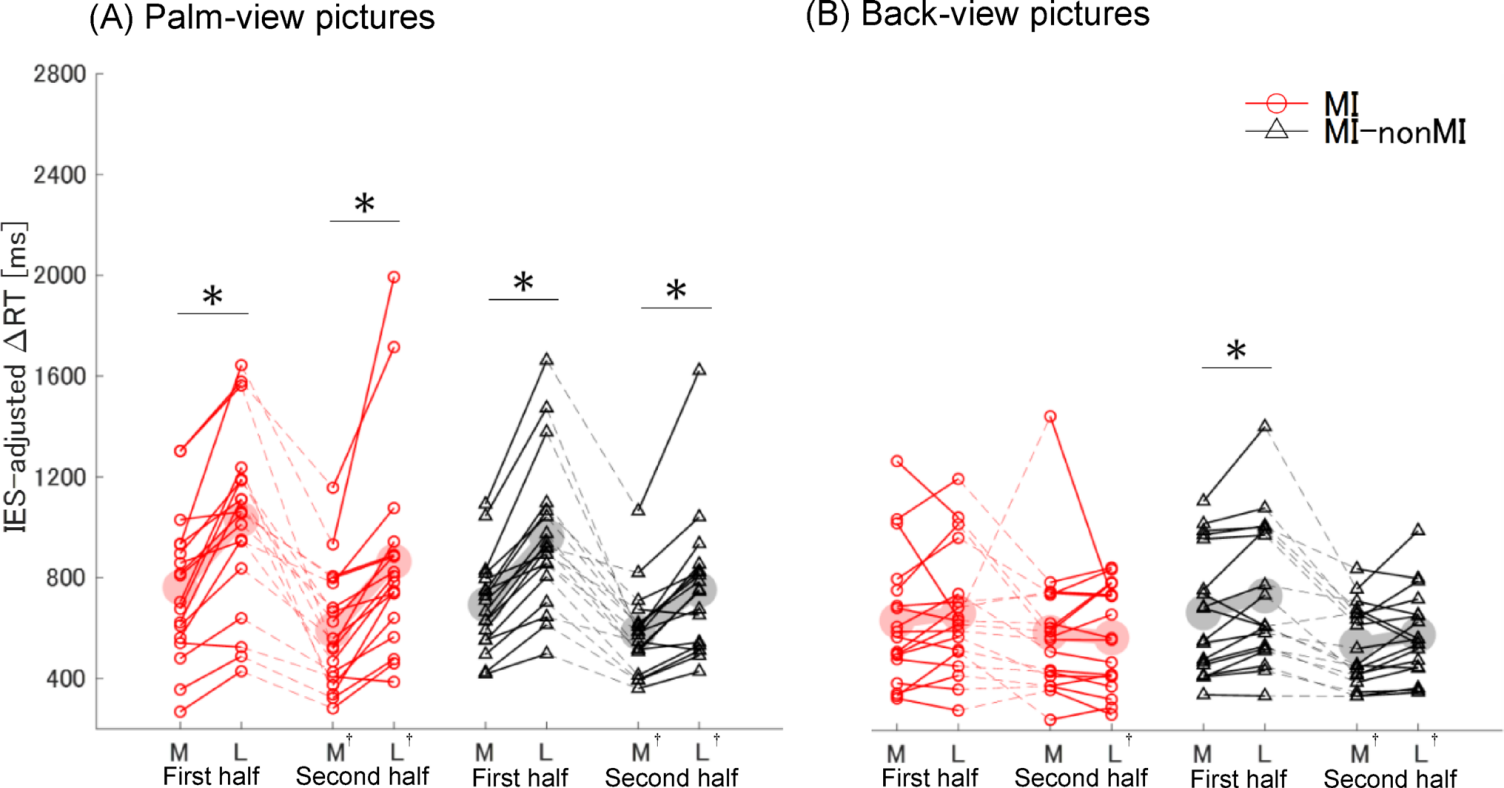
Medial–lateral (ML) effects in palm-view (**A**) and back-view (**B**) pictures. Graphs display inverse efficiency score (IES)-adjusted ΔRT values for medially (M) and laterally (L) oriented pictures, separated into first and second halves (mean of 16 trials for each half per participant). Each dot represents one participant; light-colored markers indicate group means. Red circle, MI group (participants who performed the task using MI); Black triangle, MI–nonMI group (participants who switched from MI to nonMI). For palm-view pictures (**A**), laterally oriented pictures consistently showed longer IESs than medially oriented pictures in both groups and both halves. For back-view pictures (**B**), the ML effect was observed only in the first half for the MI–nonMI group. *p < 0.05, significant difference between M and L; ^†^*p* < 0.05, second half significantly shorter than in first half

Table 2 presents the results of the three-way ANOVA conducted on the raw IESs (strategy × period × orientation) for the palm-view and back-view pictures, reporting the three-way interaction, two-way interactions, and main effects. By contrast, Fig. 3 displays individual participant data stratified consistently across all conditions and illustrates the results of the follow-up tests, specifically the simple-simple main effects, with significant differences indicated by asterisks, regardless of whether the corresponding interactions were significant.

**Table 2:** Results of the ANOVA for ΔRT (IES)

	*F*	*p*	*η2p*
Palm-view pictures			
3-way interaction (Strategy × Period × Orientation)	*F*_1,34_ = 6.65	< 0.05	0.16
2-way interaction (Period × Orientation)	*F*_1,34_ = 6.21	< 0.05	0.15
2-way interaction (Strategy × Orientation)	*F*_1,34_ = 0.78	0.38	0.02
2-way interaction (Strategy × Period)	*F*_1,34_ = 0.02	0.90	0.00
Main effect (Orientation)	*F*_1,34_ = 70.91	< 0.01	0.68
Main effect (Period)	*F*_1,34_ = 29.79	< 0.01	0.47
Main effect (Strategy)	*F*_1,34_ = 0.06	0.57	0.02
Back-view pictures			
3-way interaction (Strategy × Period × Orientation)	*F*_1,34_ = 0.32	0.58	0.01
2-way interaction (Period × Orientation)	*F*_1,34_ = 3.37	0.08	0.09
2-way interaction (Strategy × Orientation)	*F*_1,34_ = 1.30	0.26	0.04
2-way interaction (Strategy × Period)	*F*_1,34_ = 1.75	0.20	0.05
Main effect (Orientation)	*F*_1,34_ = 1.94	0.17	0.05
Main effect (Period)	*F*_1,34_ = 16.91	< 0.01	0.33
Main effect (Strategy)	*F*_1,34_ = 0.06	0.81	0.00

ΔRT (response time − motor generation response time) was adjusted using the inverse efficiency score (IES). Orientation, medial (hands facing inward) vs. lateral (hands facing outward); Period, first half vs. second half (first 16 trials vs. last 16 trials [total of 32 trials]); Strategy, MI vs. MI–nonMI groups

For the palm-view pictures, the three-way ANOVA revealed a significant interaction among strategy, period, and orientation (*p* < 0.05). Subsequent two-way ANOVAs were therefore performed separately for the MI and MI–nonMI groups. In the MI group, the interaction between period and orientation was not significant. Accordingly, we examined the overall effect of period (first vs. second half) and the overall effect of orientation (medially vs. laterally) without dividing the data by the other factor. The IESs were significantly shorter in the second half than in the first half of the trials (*p* < 0.01, *F*_1,34_ = 15.61), and that the IESs for laterally oriented hands were significantly longer than those for medially oriented hands (*p* < 0.01, *F*_1,34_ = 43.29). In contrast, in the MI–nonMI group, the interaction between period and orientation was significant (*p* < 0.01, *F*_1,34_ = 12.85). We therefore further examined the simple–simple main effects, i.e., within this group we tested (1) whether laterally oriented hands differed from medially oriented hands separately in the first and second halves, and (2) whether the first and second halves differed separately for medially and laterally oriented hands. Regarding orientation, the IESs for laterally oriented hands were significantly longer than those for medially oriented hands in both the first and second halves (both *p* < 0.01, *F*_1,34_ = 45.91 and *F*_1,34_ = 12.92, respectively). Regarding period, the IESs were significantly shorter in the second half than in the first half for both medially and laterally oriented hands (both *p* < 0.01, *F*_1,34_ = 8.36 and *F*_1,34_ = 17.22, respectively).

For the back-view pictures, ANOVA revealed no significant three-way interaction among strategy, period, and orientation, nor were any of the two-way interactions among these factors significant. Among the main effects, only period was significant (*p* < 0.01), with the IESs being significantly shorter in the second half than in the first half of the trials. Although no significant interactions were found, we conducted follow-up analyses to maintain consistency with the palm-view analysis, where such effects were examined after the significant three-way interaction. Specifically, we tested the simple–simple main effects (i.e., examining the effect of orientation within each level of period, and the effect of period within each level of orientation). Regarding period, for the MI group, the IESs for laterally oriented hands were significantly shorter in the second half than in the first half (*p* < 0.05, *F*_1,34_ = 7.23). For the MI–nonMI group, the IESs were significantly shorter in the second half than in the first half for both medially and laterally oriented hands (medial, *p* < 0.01, *F*_1,34_ = 9.00; lateral, *p* < 0.01, *F*_1,34_ = 18.19). Regarding orientation, for the MI–nonMI group in the first half of the trials, the IESs for laterally oriented hands were significantly longer than those for medially oriented hands (*p* < 0.05, *F*_1,34_ = 4.64).

**Fig. 4:**
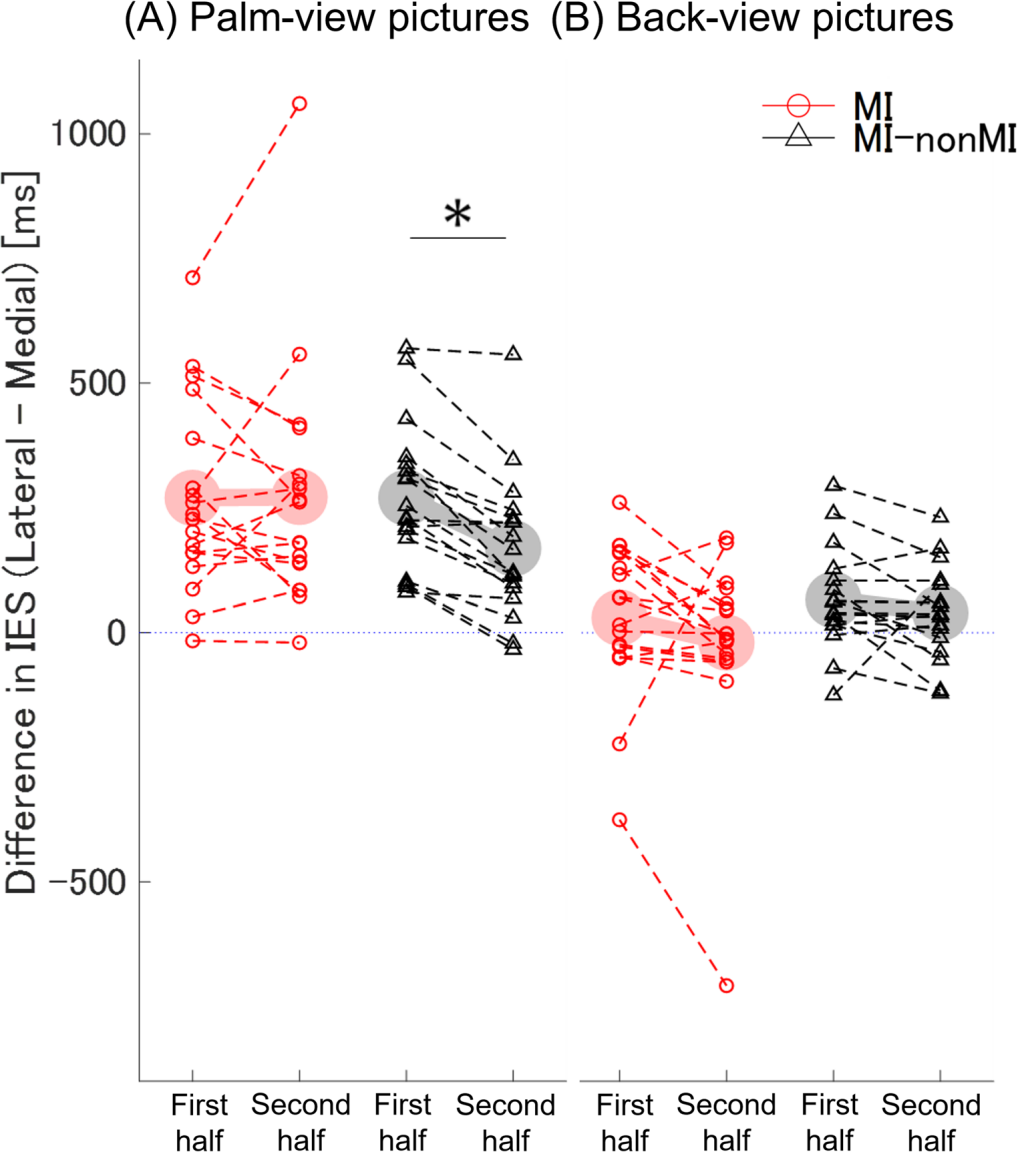
Difference in ΔRT between laterally and medially oriented hands. The difference value was calculated by subtracting the inverse efficiency score (IES) of the medially oriented hand from that of the laterally oriented hand. MI, motor imagery throughout; MI–nonMI, switched from MI to nonMI. **A** Palm-view: no significant difference between halves in the motor imagery (MI) group (red circle), but the MI–nonMI group (black triangle) showed a shorter difference in the second half. **B** Back-view: no significant difference across halves and either group. Light-colored markers indicate mean values. *p < 0.05 (first half vs. second half)

Table 3 shows the results of the two-way ANOVA conducted on the ML effect (i.e., the difference between lateral and medial IESs), with strategy (MI vs. MI–nonMI) and period (first half vs. second half) as factors. For the palm-view pictures, two-way ANOVA revealed a significant interaction between strategy and period. Subsequent simple main effects analysis indicated that the IESs were significantly shorter in the second half compared with the first half only in the MI–nonMI group (Fig. 4A). For the back-view pictures, two-way ANOVA revealed no significant interaction between strategy and period, nor significant main effects of period or strategy (Fig. 4B). Because both factors had only two levels, reporting the F-values was sufficient to indicate whether differences existed.

**Table 3:** Results of two-way ANOVA of the difference in IESs between laterally and medially oriented hand pictures

	*F*	*p*	*η2p*
Palm-view pictures			
Interaction (Strategy × Period)	*F*_1,34_ = 6.65	0.01	0.16
Simple main effect			
MI strategy (first half vs. second half)	*F*_1,34_ = 0.00	0.95	0.00
MI–nonMI strategy (first half vs. second half)	*F*_1,34_ = 12.85	< 0.01	0.27
Back-view pictures			
Interaction (Strategy × Period)	*F*_1,34_ = 0.32	0.58	0.01
Main effect (first half vs. second half)	*F*_1,34_ = 3.38	0.08	0.09
Main effect (MI vs. MI–nonMI)	*F*_1,34_ = 1.30	0.26	0.04

The magnitude of the ML effect was calculated as the difference in inverse efficiency scores (IESs) between laterally and medially oriented hands (lateral−medial IES). MI, motor imagery throughout; MI–nonMI, switched from MI to nonMI; Period, first half vs. second half (16 trials each); Strategy, MI vs. MI–nonMI groups

## Discussion

In the self-reported explicit strategy, most participants consistently performed the back-view picture trials without using the MI strategy, regardless of trial repetition. In contrast, half of the participants changed their strategy for the palm-view pictures from the MI strategy to the nonMI strategy, including the VI strategy. Specifically, participants in the MI–nonMI group initially relied on imagining their own hand (or shoulder) movements and later switched to the nonMI strategy, which involved mentally rotating the hand picture or using cues, such as the orientation of the index finger and thumb, to make their judgments. Studies suggest that back-view pictures tend to elicit the VI-like strategy, as participants may rely on the orientation of the index finger and thumb to determine laterality (Lust et al. 2006; Conson et al. 2021). The participants in this study who used finger orientation as a cue may have performed the task by identifying specific features that facilitated discrimination, rather than by mentally rotating the entire picture. In the MI–nonMI group, the difference in RT (IESs) between laterally and medially oriented palm-view pictures significantly decreased with trial repetition. This result also suggests that the shift from the MI strategy to the nonMI strategy was not only self-reported, but also reflected in the behavioral data. In contrast, the ML effect, characteristic of the MI strategy, persisted into the second half of the task in the MI–nonMI group. This result may indicate that the timing of the strategy shift varied among participants, with some likely switching strategies only in the final phase of the experiment. With a sufficiently large sample size, dividing the trials into more granular phases rather than just two halves (first and last) would allow for a more detailed analysis of when the strategy shift occurred. An important feature of the present study was the relatively large number of trials (512 in total, with each of the 16 hand pictures presented 32 times), which enabled us to detect such temporal changes in strategy. Many previous HLJ studies (Ionta et al. 2007; Saimpont et al. 2009; Nagashima et al. 2021) employed fewer trial repetitions. Given their design, it was not necessary to assume potential changes in strategy within individuals, and such shifts were therefore not considered. This difference suggests that some of the individual differences in strategy reported in earlier studies may partly reflect unobserved temporal shifts that occurred during task performance but remained unnoticed due to the smaller number of trials. Moreover, not only the number of repetitions but also the diversity of hand pictures presented is likely to influence strategy use. Limited picture variation may encourage participants to adopt pattern-matching strategies, such as relying on the “L-shape” formed by the index finger and thumb to discriminate laterality. While such approaches are categorized as nonMI, their likelihood may increase with repeated exposure to a restricted set of stimuli, whereas greater variability in hand pictures may help sustain the use of MI across trials.

In the HLJ task, palm-view pictures are generally considered to be judged using an implicit MI strategy (de Lange et al. 2006; Zapparoli et al. 2014; Conson et al. 2017). However, a study measuring event-related potentials suggested that the strategy may vary depending on the direction of the palm-view pictures; specifically, it was been shown that laterally oriented pictures with large angular deviations from the upright position may involve the nonMI strategy, in which the picture is mentally rotated (ter Horst et al. 2012). Performance might involve both the MI and nonMI strategies (Mochizuki et al. 2019). In the MI–nonMI group of the present study, participants explicitly reported shifting from the MI strategy to the nonMI strategy when judging palm-view pictures. However, the presence of the ML effect in the second half of the trials suggests that they may have continued to use the implicit MI strategy. Interestingly, in this group, the ML effect was also observed for back-view pictures, even though the participants explicitly reported using the nonMI strategy. This result suggests that the implicit MI strategy may have been used regardless of whether the picture depicted the palm or the back of the hand during a period of strategy re-evaluation.

The physical position of the hands during a task can serve as a reference for MI (Shenton et al. 2004; de Lange et al. 2006; Ionta et al. 2007). In the present study, although the participants’ hands were covered to avoid visual cues, they were positioned with the backs of the hands facing upward. Therefore, when back-view pictures were presented, the participants may have discriminated laterality by mentally aligning the visualized hand with their own perceived hand posture. Specifically, they may have rotated the stimulus image in mind to match their actual hand position, or alternatively relied on heuristics such as salient visual cues (e.g., thumb orientation, the L-shape of the index finger and thumb) for direct pattern matching. In line with recent discussions (Dahm and Draxler 2023), such heuristic approaches can be regarded as distinct from visual imagery. However, in this study we classified both VI and heuristic approaches together as nonMI strategies. This interpretation is consistent with prior findings that back-view pictures are often solved without engaging MI (ter Horst et al. 2010; Conson et al. 2021). The adopted strategy might differ depending on the response methods, such as pressing buttons with the palms facing upward or using foot switches while keeping the hands behind the back.

In clinical rehabilitation, training options for promoting motor functional recovery in the paralyzed limbs of patients with severe hemiplegic stroke are limited, and clinical attention is often directed toward acquiring ADL functions using the non-paralyzed limb. Because MI does not require actual movement, its application in rehabilitation for patients with severe motor deficits has been anticipated (Ji et al. 2021; Villa-Berges et al. 2023). Neuroimaging studies have shown that motor-related brain regions are activated not only during actual movement but also during MI (Hanakawa et al. 2003). In particular, first-person MI activates the primary motor cortex, supplementary motor area, and premotor cortex (Jackson et al. 2001), suggesting the potential to stimulate motor-related cerebral function even in the absence of overt movement. Upper limb function was improved following a rehabilitation program incorporating MI induced by the HLJ task and mirror therapy in patients with chronic stroke (Ji et al. 2021). Additionally, the presence of the ML effect during the HLJ task in patients with stroke has been interpreted as evidence of MI engagement (Harada et al. 2016). Given that implicit MI is elicited during left–right judgments of palm-view pictures in the HLJ task, it is considered a potential option for rehabilitation. However, the present study reveals that performance strategies may shift over repeated trials of the HLJ task, which is a critical consideration for its clinical application. Notably, back-view pictures are reported not to elicit MI (ter Horst et al. 2010), whereas palm-view pictures and those presented from alternative viewpoints (e.g., thumb side, little finger side) are more likely to induce MI (de Lange et al. 2006; Ionta and Blanke 2009). In addition to trial repetition, variations in hand pictures should also be considered. Limited picture variation may prompt participants to rely on pattern matching rather than engaging in MI. Therefore, task designs using trial-unique hand pictures may be required. For example, using hand pictures with combinations of finger flexion and extension (Takeda et al. 2010), or increasing the range of visual perspectives (e.g., fingertip views, oblique palm angles), may promote MI induction. To improve the HLJ task for clinical use, picture variation aimed specifically at facilitating MI strategies is likely necessary.

## Data Availability

No datasets were generated or analysed during the current study.
